# New Insights on Hydration Monitoring in Elderly Patients by Interdigitated Wearable Sensors

**DOI:** 10.3390/s25227081

**Published:** 2025-11-20

**Authors:** Leila Es Sebar, Stefano Bonaldo, Loredana Cristaldi, Lara Franchin, Sabrina Grassini, Leonardo Iannucci, Luca Lombardo, Chiara Mineo, Andrea Neviani, Lorenzo Restelli, Isabella Sannino, Sarah Tonello, Cesare Svelto

**Affiliations:** 1 Dipartimento di Elettronica, Informazione e Bioingegneria, Politecnico di Milano, Piazza Leonardo da Vinci 32, 20133 Milano, Italy; loredana.cristaldi@polimi.it (L.C.); lorenzo2.restelli@mail.polimi.it (L.R.); cesare.svelto@polimi.it (C.S.); 2 Dipartimento di Ingegneria dell’Informazione, Università degli Studi di Padova, Via Gradenigo 6b, 35131 Padova, Italy; stefano.bonaldo@unipd.it (S.B.); franchinla@dei.unipd.it (L.F.); andrea.neviani@dei.unipd.it (A.N.); 3 Dipartimento di Scienza Applicata e Tecnologia, Politecnico di Torino, Corso Duca degli Abruzzi 24, 10129 Torino, Italy; sabrina.grassini@polito.it (S.G.); chiara.mineo@polito.it (C.M.); isabella.sannino@polito.it (I.S.); 4 Dipartimento di Elettronica e Telecomunicazioni, Politecnico di Torino, Corso Duca degli Abruzzi 24, 10129 Torino, Italy; luca.lombardo@polito.it; 5 Dipartimento di Ingegneria Civile, Architettura, Territorio, Ambiente e di Matematica, Università degli Studi di Brescia, Piazza del Mercato, 15, 25121 Brescia, Italy; s.tonello@unibs.it

**Keywords:** interdigitated electrodes, hydration monitoring, electrochemical impedance spectroscopy, wearable devices, inkjet printing, health monitoring, elderly monitoring, biosensors, telemedicine, metrological characterization

## Abstract

The progressive aging of the population requires reliable, non-invasive, and real-time tools to monitor hydration, prevent dehydration-related complications, and promote active aging in elderly patients. Wearable sensors based on interdigitated electrodes (IDEs) and on Electrochemical Impedance Spectroscopy (EIS) represent a promising tool thanks to their miniaturization, sensitivity to dielectric variations with humidity, and compatibility with flexible substrates. This study reports the design, fabrication, and metrological characterization of inkjet-printed IDEs for skin hydration monitoring, as a building block of a multisensor wearable device. IDEs were fabricated on polyimide substrates using silver nanoparticle-based ink. Their characterization involved the following: (i) morphological evaluation by scanning electron microscopy; (ii) EIS measurements in KCl solutions, leading to developing a regression model to correlate impedance with salt concentration; (iii) in vitro EIS validation on agar gel samples, which demonstrated a robust linear relationship between the impedance phase shift at 199.5 Hz and water loss, with consistent sensitivity values across sensors. The results confirm the feasibility of IDEs for hydration monitoring, identifying optimal frequency ranges and validating regression models. These findings represent a critical step toward the development of multisensor wearable devices for elderly monitoring, enabling decentralized and continuous health monitoring to improve healthcare sustainability and telemedicine.

## 1. Introduction

Telemedicine is a promising tool for supporting active aging, which represents a priority for the European Union. The fraction of individuals over 65 years old is expected to increase by 2050 from the current 20% to 30% in Europe, and from 23.5% to nearly 35% in Italy (sources: EUROSTAT and ISTAT). This demographic shift will strongly affect the future design of healthcare systems, due to the growing demand for professional services, social care, and medical assistance. In this scenario, preventing healthcare system saturation and improving elderly quality of life are strictly linked to the ability to monitor health conditions not only in hospitals but also through autonomous, user-friendly devices. This represents a crucial step toward the establishment of a widespread decentralized home-care network that can promote active aging and reduce hospitalizations [1].

Beyond standard vital signs, alterations in water balance and in electrolyte and metabolite levels in blood are among the most relevant health indicators in elderly patients. Dehydration is common in elderly patients due to several factors, such as reduced thirst perception, neurodegenerative diseases leading to reduced fluid intake, renal or cardiac disorders, and loss of muscle mass. When dehydration remains uncompensated, it may result in delirium, tachycardia associated with hypotension, and, in worst-case scenarios, life-threatening complications [2]. In such cases, clinically assisted nutrition may be necessary to ensure adequate fluid and nutrient intake [3,4].

The diagnosis of dehydration is challenging due to the diversity of parameters involved. Current practices rely on indirect, non-physiological indicators and on clinical evaluations that involve both qualitative and quantitative approaches [5,6]. Hydration is typically monitored using parameters such as urine osmolality, urine color, urine specific gravity, body weight variation, urine-specific gravity, and blood analysis [6,7,8,9,10,11]. However, urine color is a nonspecific marker, and in many cases, patients may not produce enough urine for reliable assessment. Other diagnostic methods that can identify dehydration are often invasive, inconvenient, or uncomfortable, and they lack the ability to provide real-time alerts to prevent complications. Moreover, most quantitative techniques require advanced instrumentation, laboratory analyses, specialized medical staff, and controlled environments, making them not suitable for everyday use [5,11,12]. Consequently, such an assessment is usually performed upon hospital admission, when dehydration may already be advanced. In addition, electrolyte and metabolite imbalances (e.g., hypernatremia, hyperglycemia, hyperlactatemia) are crucial biomarkers of common age-related conditions such as renal dysfunction, diabetes, and cardiovascular disease [13,14]. To the best of the Authors’ knowledge, nowadays there is no real-time, non-invasive, and simple method to measure hydration levels in the elderly [11].

Therefore, simultaneous monitoring of these parameters via a multisensor wearable device is a promising strategy for preventing critical events [15]. Wearable technologies have emerged as promising tools for decentralized healthcare and telemedicine, since they enable real-time tracking of physiological parameters through miniaturized, non-invasive sensors, with adequate accuracy, ease of use, and comfort [16,17,18]. Although recent optical methods exploiting speckle pattern analysis and machine learning have achieved remarkable results in non-contact analysis of turbid or biological samples, their suitability for continuous, on-skin monitoring is still limited. Most current wearable devices, therefore, focus on either electrolyte/metabolite monitoring or body composition/skin hydration assessment [18,19,20]. Some studies have reported the development of flexible humidity and moisture sensors for environmental or epidermal applications. However, it is important to distinguish between hydration, i.e., the physiological water content within the skin and underlying tissue, and humidity/moisture, which refers to the amount of water in the surrounding air or on the surface. Flexible humidity sensors, such as those based on porous ionic membranes [21] and paper-based platforms [22], have proved sensitivity for detecting atmospheric or surface moisture. Yet, they primarily respond to ambient relative humidity rather than to water content of the skin and thus assessment of skin hydration. In contrast, impedance-based methods can detect dielectric changes within biological tissues, reflecting their hydration state and enabling real-time, on-skin monitoring relevant to healthcare and telemedicine applications. Indeed, electrochemical methods, such as potentiometry and chronoamperometry, are reliable approaches for electrolyte and metabolite analysis [23], with proven robustness and suitability for wearable integration across multiple applications [24]. For body composition, bioelectrical impedance analysis (BIA) is a widely adopted technique based on low-intensity AC current injection through four electrodes and voltage measurement; impedance variations are then correlated with patient body composition [25,26]. Miniaturized BIA-based wearable devices have been proposed for both body composition and indirect monitoring of heart rate or blood pressure [27,28]. Indeed, impedance-based techniques have attracted growing interest, since they provide a versatile approach for monitoring hydration and tissue composition [29]. In particular, interdigitated electrodes (IDEs) stand out as a powerful sensing solution, owing to their miniaturization capability, high sensitivity to local dielectric changes, and compatibility with flexible substrates for skin-contact applications. Previous studies have demonstrated their potential application to skin hydration monitoring [30], yet systematic characterization in conditions relevant to elderly care remains limited.

In this context, the project “MuSe: Multi-Sensor Wearable Device for Telemedicine” (funded by the Italian Ministry of University and Research under the PRIN 2022 program) aims at the development of an autonomous wearable system for hydration, metabolic, and general health monitoring in elderly populations through a multisensor approach. The ultimate project goal is to provide an effective, comfortable, and scientifically reliable tool capable of seamless integration into elderly daily life, thereby improving the quality of life and enhancing the sustainability of healthcare systems.

Within the scope of this project, this paper is focused specifically on the design and characterization of interdigitated electrodes for skin hydration monitoring, as a fundamental building block of the multisensor device.

While the elderly represent the main use case motivating this study, given their higher susceptibility to dehydration due to physiological and behavioral factors, the proposed sensing approach is broadly applicable to other user groups and clinical contexts requiring non-invasive hydration assessment.

## 2. Interdigitated Electrodes for Hydration Monitoring

Interdigitated electrodes are widely employed in electrochemical and bioimpedance applications due to their ability to detect small changes in the dielectric properties of the surrounding medium. Their geometry, consisting of parallel interleaved fingers, provides a large effective electrode surface area while maintaining compact dimensions, making IDEs particularly suitable for miniaturized and wearable formats [31,32,33,34]. IDEs offer significant advantages over traditional rigid or gel-based electrodes by ensuring conformable contact with the skin, thereby reducing issues such as inconsistent contact, motion artifacts, and skin irritation, which are crucial for long-term, continuous monitoring, particularly in elderly care [6,30].

In hydration monitoring, IDEs can be employed in combination with Electrochemical Impedance Spectroscopy (EIS). EIS offers many advantages, being non-invasive and based on the application of a small electrical stimulus, and it can be employed to track changes in skin and tissue hydration due to its high sensitivity to the water content [35]. In addition, due to its relative simplicity, many portable and low-cost instruments based on EIS have been developed, which can be integrated in a wearable device to perform impedance measurements [36]. Indeed, as water content fluctuates, it alters the skin’s electrical conductivity and permittivity, which these sensors are designed to measure. The fringing electric field between the interdigitated electrodes penetrates the upper layer of the skin, allowing for direct measurement of skin impedance [32,35,37].

Several fabrication strategies have been explored, including inkjet printing of conductive nanoparticle inks, aerosol jet deposition, and thin-film photolithography, enabling integration onto flexible polymeric substrates such as polyimmide and polyethylene terephthalate. These approaches ensure conformability with the skin surface while preserving mechanical robustness under bending and long-term use [38]. Performance studies have shown that IDEs can provide sensitive response to ionic concentration variations in fluids, as well as to hydration changes in tissue-mimicking phantoms, such as agar gels [30,33,37] or absorbing skin-mimicking patches [31]. Critical parameters affecting sensitivity include electrode spacing, finger width, and surface treatment, which determine the penetration depth of the fringing electric field and, consequently, the interaction volume with the target medium. IDEs have also demonstrated good repeatability and stability, essential for wearable healthcare applications [30,32].

Despite these advantages, gaps remain in the systematic metrological characterization of IDEs for real-world hydration monitoring. Most reported studies focus on controlled laboratory conditions [31] or athletic monitoring [39,40], whereas elderly-oriented telemedicine applications demand additional requirements, including robustness to inter-individual variability, long-term stability, comfort, and integration within wireless wearable platforms [41]. Addressing these aspects is crucial to translating IDE-based sensing into reliable tools for decentralized healthcare. In addition, although interdigitated electrodes and EIS have been exploited to measure ionic concentration and skin hydration, most studies either present proof-of-concept demonstrations or focus on single aspects (electrode design, phantom construction, or signal processing) without delivering a complete metrological pathway from electrode morphology to calibrated sensing in tissue-mimicking material. To fill this gap, we present the design, fabrication, and in vitro characterization of printed IDEs optimized for skin hydration monitoring, as a step toward their integration into the multisensor wearable device and in vitro trials.

In particular, the paper presents a combined study that (i) characterizes the printed IDE morphology, (ii) establishes a predictive regression model correlating impedance to salt concentration in known potassium chloride (KCl) solutions, and (iii) validates the sensors on agar phantoms undergoing controlled dehydration to demonstrate correlation between impedance phase shift and water loss thus proving the potential application for dehydration monitoring on patients. The adopted interdigitated layout ensures high sensitivity to changes in the skin’s electrical properties, such as dielectric permittivity and electrical conductivity, which are caused by small local variations of water concentration in skin tissues [31]. Furthermore, the use of a flexible polyimide substrate provides complete conformability of the sensor with the skin, improving the comfort for the end-user and meeting the requirements for future wearable applications. Although the present work focuses on the design and metrological characterization of the IDEs, this study represents a crucial step toward integrating the sensor into a fully wearable, multisensor platform for real-time hydration monitoring.

## 3. Materials and Methods

This section describes the materials, their characterization, and the EIS setup used for the measurements in saline solutions and on agar samples.

The sensor performance was evaluated by monitoring impedance modulus and phase shift variations under controlled changes in electrolyte concentration and hydration level. With this goal, two complementary experimental protocols were implemented: calibration in KCl solutions to assess sensitivity to ionic concentration, and in vitro validation on agar gel phantoms undergoing controlled dehydration to simulate different hydration states.

### 3.1. Printed Interdigitated Electrodes for Skin Hydration Monitoring

**Figure 1:**
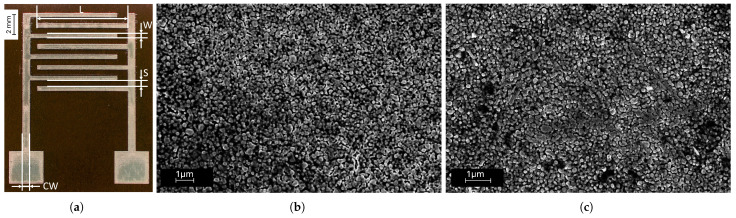
(**a**) Interdigitated electrode (marker = 2 mm): electrode length *L* = 9.0 mm, electrode width *W* = 0.45 mm, spacing *S* = 0.6 mm, collector width *CW* = 0.8 mm); scanning electron microscopy images of the silver layer acquired before (marker = 1 μm) (**b**) and after (marker = 1 μm) (**c**) the sensor was used.

The interdigitated electrodes under study were fabricated using inkjet printing, a non-contact technique offering high flexibility in terms of substrate, ink selection, and achievable geometries [38]. The printer employed was a Dimatix DMP 2850 (FUJIFILM, Santa Clara, CA, USA), using a silver nanoparticle-based ink (Sicrys I40DM-106, PVNanocell, Migdal Ha’Emek, Israel) with an average particle size of 30 nm suspended in a diethylene glycol monomethyl ether (DGME) solvent, specifically optimized for digital inkjet printing. The nanostructured nature of the ink provides enhanced electrical properties, improved substrate adhesion, and superior oxidation resistance. The ink was deposited on a 50 μm polymmide film (Kapton, American Dupont Company, Wilmington, DE, USA). This substrate was chosen to maximize repeatability and robustness of the printing process while ensuring comfort for in vivo applications on skin. Polyimide is characterized by excellent thermal, electrical, mechanical, and chemical stability, enabling optimal adhesion during printing and high mechanical integrity after curing. The substrate thickness was selected to enhance conformability with the skin surface and to avoid localized adhesion losses that could alter surface contact impedance and measurement accuracy. After printing, the electrodes were cured at 250 °C for 30 min. The electrode geometry was a rectangular interdigitated layout, consistent with designs reported in previous works [42]. The design parameters were as follows: electrode length *L* = 9.0 mm, electrode width *W* = 0.45 mm, spacing *S* = 0.6 mm, and collector width *CW* = 0.8 mm, as shown in Figure 1a. The active sensing area of each interdigitated structure was 0.856 cm^2^.

### 3.2. Scanning Electron Microscopy (SEM) Characterization

The morphological characterization of the sensor surface was carried out using a Field Emission Scanning Electron Microscope (FESEM, Supra 40, Zeiss, Jena, Germany). Images of the sensors (Figure 1b) were acquired using the secondary electron detector, with an accelerating voltage of 5 kV and an electron beam aperture of 30 µm. The sensors were characterized before and after the test in both saline solutions and on agar samples.

### 3.3. Electrochemical Impedance Spectroscopy Characterization

EIS was employed with a twofold objective: First, the IDEs were characterized in saline environments to develop a predictive regression model correlating impedance modulus to salt concentration in known KCl solutions. Subsequently, the sensors were validated on agar phantoms subjected to controlled dehydration, in order to investigate the correlation between impedance phase shift and water loss; in the end, demonstrating their potential application for monitoring dehydration in patients.

For both measurements, in solutions and on agar samples, EIS measurements were carried out using a PalmSens4 (PalmSens^®^ BV, Houten, The Netherlands) electrochemical interface, with a two-electrode configuration and a sine-wave excitation of 6 mV amplitude. Frequency sweeps were performed from $10^{5}$ Hz to 1 Hz, with 5 points acquired per frequency decade (26 points in total).

#### 3.3.1. Electrochemical Impedance Measurements in Saline Solutions

**Figure 2:**
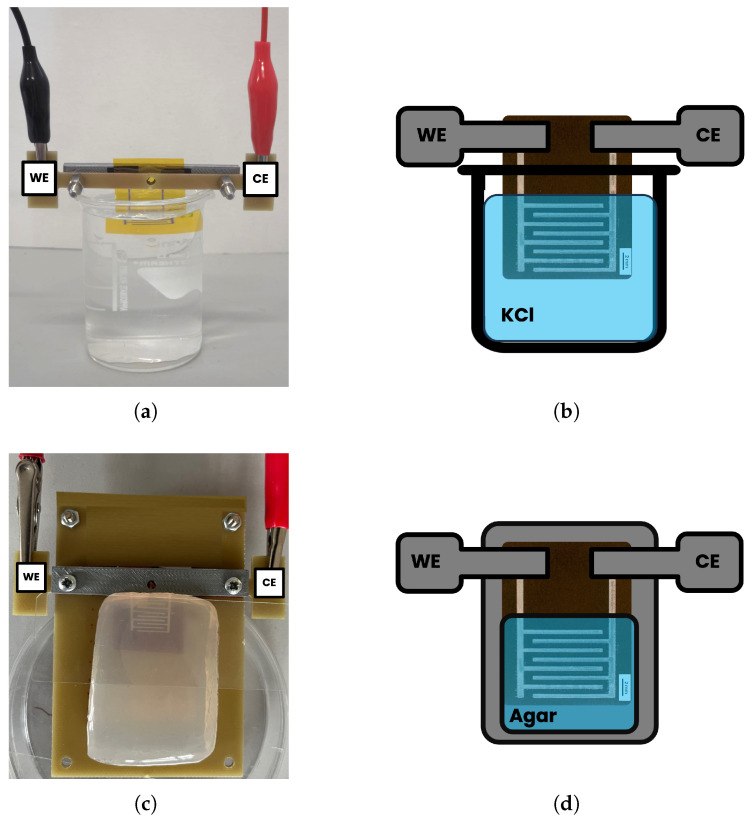
(**a**) Acquisition setup for electrochemical impedance measurements in saline solutions and (**b**) schematic representation of the setup (WE: working electrode, CE: counter electrode, KCl: potassium chloride). (**c**) Acquisition setup for electrochemical impedance measurements on agar samples and (**d**) schematic representation of the setup (WE: working electrode, CE: counter electrode).

EIS measurements were performed in saline solutions to correlate impedance values with salt concentration. The measurements were performed placing the electrodes inside the saline solution (Figure 2a); a schematic representations of the experimental contacting setups is reported in Figure 2b. Solutions were prepared by mixing deionized water and KCl (Sigma-Aldrich, St. Louis, MO, USA) at concentrations of 0.25, 0.50, 1.00, 1.50, 2.00, 3.00, and 4.00 mmol/L with uncertainty better than 0.01 mmol/L. These concentrations were selected for IDEs characterization since they fall within the typical physiological range reported for potassium ions in human sweat [43]. For this test, 3 sensors were used. EIS spectra were acquired sequentially by immersing each sensor in the solutions with increasing salt concentrations (i.e., 0.25, 0.50, 1.00, 2.00, and 4.00 mmol/L), starting from 0.25 mmol/L. For each concentration, three EIS spectra of measured impedance modulus and phase were repeatedly acquired for each sensor. To monitor the variation of solution resistance as a function of the KCl concentration, the value of the impedance modulus at the frequency of 15.8 kHz (the closest available value to 10 kHz in the frequency scan) was recorded. Indeed, in this frequency region (above 10 kHz), the impedance response presents a predominantly resistive behavior, as proved by the phase approaching 0°. Thus, the impedance modulus is directly correlated to the solution resistance and so the electrolyte concentration. Therefore, this frequency was chosen to represent the bulk solution resistance and to construct the calibration plot correlating impedance with KCl concentration. This criterion ensured a consistent and physically meaningful comparison across all measurements. Then, for each concentration, average values and standard deviations over 3 sensors were calculated. The absolute impedance values were normalized to the sensor’s active surface area, to obtain impedance values not affected by the electrode dimension. In particular, since the EIS measured impedance depends inversely on the sensor’s area, the normalization was performed by multiplying each measured impedance value by the active sensing area of the interdigitated electrode (0.856 cm_2_). This step removes the geometric influence of electrode size, enabling comparison among different IDEs. Consequently, the normalized impedance represents the area-specific impedance, conventionally expressed in Ω·cm^2^, as in standard electrochemical analyses [44]. These values were then plotted as a function of KCl concentration, and a non-linear regression was applied to obtain the calibration curve describing the relationship between impedance modulus and electrolyte concentration. To assess the predictive capability of this model, two additional concentrations (1.50 and 3.00 mmol/L) were measured under the same conditions, and the corresponding concentrations were estimated from the regression curve.

#### 3.3.2. Electrochemical Impedance Measurements on Agar Samples

The potential application for hydration monitoring was evaluated in vitro using agar gel samples, selected as tissue-mimicking materials, containing different water volumes to simulate skin hydration behavior. Agar is a material widely employed to simulate skin and soft tissues in in vitro testing due to its dielectric properties, which are comparable to those of biological tissues [30,45,46]. Agar gels were prepared by dissolving agar powder (12.0 g/L, concentration chosen according to the indications from the producer) (Sigma-Aldrich, St. Louis, MO, USA) and KCl (10.0 mmol/L) (Sigma-Aldrich, St. Louis, MO, USA) in deionized water, and placing the mixture on a hot plate at 90 °C, continuously stirring and yielding a homogeneous solution. The quantity of deionized water determines the water content, while sodium chloride adjusts the conductivity, and agar powder allows for self-shaping of the samples [30]. The solution was then cast into Petri dishes and cooled to room temperature. After solidification, the samples were placed in a climatic chamber at 37 °C to simulate physiological temperature.

**Figure 3:**
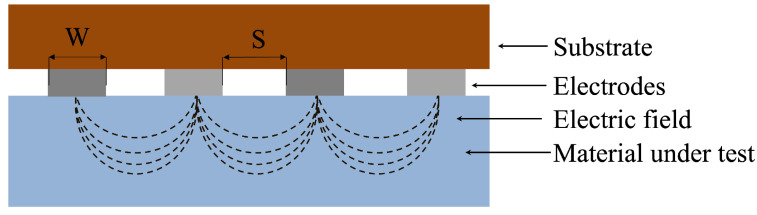
Electrochemical impedance measurements on the material under test: qualitative representation of the electric field distribution (dashed lines) in the material cross-section. Dark outer layers indicate the sensor substrate, and grey regions represent the electrodes, while the material under test is shown in blue. Image not to scale (W = electrode width and S = electrode spacing).

The EIS measurements were performed by placing the interdigitated electrodes on top of the Material Under Test (MUT), which in a real application is represented by the skin. Upon application of a small alternating voltage between the electrodes, ionic species within the MUT facilitate charge transport, and the resulting impedance measured at the electrodes reflects the combined effects of ionic conductivity, dielectric permittivity, and interfacial polarization at the electrode–material interfaces. The distribution of the electric field within the MUT depends on the local dielectric properties of the layers underlying the electrodes. Thus, any local change in these properties involves a change in the measured impedance. In particular, hydration level influences both the ionic conductivity and the dielectric permittivity leading to measurable changes in impedance magnitude and phase due to a change in the layer conductivity and the formation of a double ionic layers. Figure 3 depicts the arrangement of the measurement setup with the interdigitated electrodes placed on top of the MUT and a qualitative representation of the electric field distribution.

Water loss due to evaporation was monitored up to 150 min from the insertion in the climatic chamber, both by gravimetric measurements and by EIS using the proposed sensors. Measurements were acquired after 10, 30, 60, 90, 120, and 150 min of exposure to air in the climatic chamber. The experiment was performed using six sensors. A different agar sample was used for each sensor, and at each time point the percentage mass loss was determined gravimetrically. The percentage mass loss ($\Delta m_{\%}$) was defined as in Equation (1),

(1) $$\Delta m_{\%}=\left(m_{0}-m_{i}\right)\;\cdot \;100/m_{0}$$

where $m_{0}$ is the initial mass of the hydrated sample and $m_{i}$ is the mass after each exposure interval.

In order to identify the frequency of highest sensitivity of the phase change with respect to the water loss, the percentage phase shift ($\Delta \phi_{\%}$) normalized to the initial ϕ value at time $t_{0}$ ($t_{0}=0$ min) was computed for each sensor, across the full range of frequencies, as reported in Equation (2),

(2) $$\Delta \phi_{\%}=\left(\phi_{t_{i}}-\phi_{t_{0}}\right)\;\cdot \;100/\phi_{t_{0}}$$

where $\phi_{t_{i}}$ is the phase measured at time $t_{i}$, and $\phi_{t_{0}}$ is the initial phase value at time t=0 min.

Then, at each frequency, the relationship between $\Delta \phi_{\%}$ and the corresponding $\Delta m_{\%}$ was quantified by calculating the slope of the regression line, which reflects the sensor’s phase sensitivity at that frequency. A slope-versus-frequency profile was obtained for each sensor, and the point of highest sensitivity for each sensor was identified as the frequency at which the slope reached its maximum value.

## 4. Results and Discussion

In this section, the results of the different characterizations performed on the interdigitated electrodes are presented. First, the surface morphology analysis by SEM is discussed, followed by the results and discussion of EIS measurements performed both in saline solutions and on agar gel samples.

### 4.1. Scanning Electron Microscopy Characterization

The electrodes fabricated via inkjet printing exhibit remarkable flexibility, attributable to the reduced thickness of both the polyimmide substrate and the silver conductive film. The polymeric material was selected for its chemical stability, which ensures excellent adhesion of the conductive ink.

Looking by eye, the silver traces show a mirror-like glossy surface finish, which is preserved even after several days of exposure to air, demonstrating good oxidation resistance and stable surface properties of the conductive material.

The surface morphology revealed by the SEM images is characterized by the presence of nanometric silver aggregates, homogeneously distributed on the polymeric substrate, forming the electrode material (Figure 1). Direct comparison of the surfaces before and after sensor use indicates that the morphological structure of the aggregates remains substantially unchanged, confirming the mechanical and chemical stability of the conductive film during operation.

### 4.2. Electrochemical Impedance Measurements in Saline Solutions

**Figure 4:**
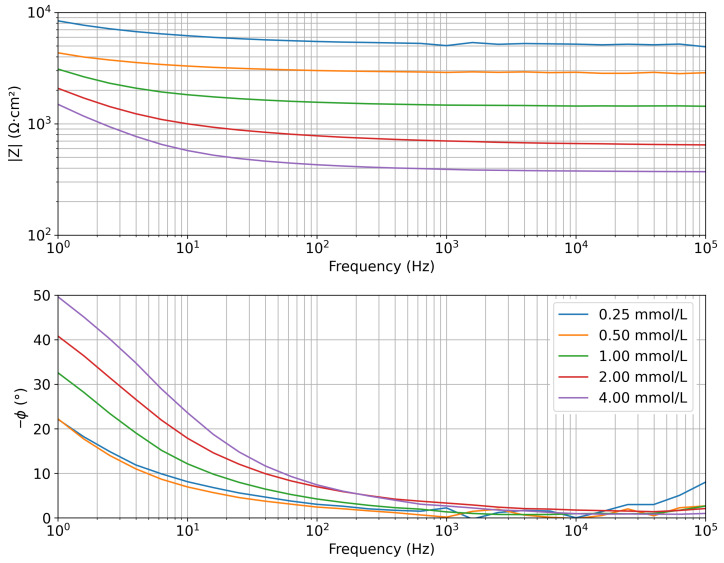
Representative spectra of modulus and phase of the measured electrochemical impedance (Bode plots) acquired in saline solutions with increasing KCl concentrations (mmol/L).

EIS measurements acquired in saline solutions with increasing KCl concentrations (0.25, 0.50, 1.00, 2.00, and 4.00 mmol/L) are reported in Figure 4 as Bode plots, showing the impedance modulus and phase.

The electrochemical system has a different response as a function of the frequency. At high frequencies (above 10 kHz), all spectra exhibit a resistive behavior, as indicated by the phase approaching 0°. In this region, the impedance modulus decreases with increasing electrolyte concentration; this is because the impedance is dominated by the solution resistance. The resistance is lower at higher KCl concentrations, consistent with the expected increase in solution conductivity. At low frequencies, a progressive increase in phase values is observed, indicating the presence of capacitive behavior associated with the electrical double layer at the electrode–solution interface.

Since the impedance spectrum at high frequencies is primarily influenced by the solution resistance, whereas the low-frequency region is dominated by the electrode–solution interface, the analysis was focused on the high-frequency region, which is relevant for measuring the salinity of the solution in which the sensor is immersed. In particular, to enable a comparative evaluation across different concentrations, the impedance modulus was extracted at a fixed frequency of 15.8 kHz (the first value in the frequency decade starting at 10 kHz), sufficiently high to predominantly reflect the resistive response of the solution. Mean values and standard deviations were then calculated for each concentration and each sensor. In addition, the mean values for each concentration across the whole dataset of the 3 sensors and the corresponding standard deviation were computed, and they are listed in Table 1.

**Figure 5:**
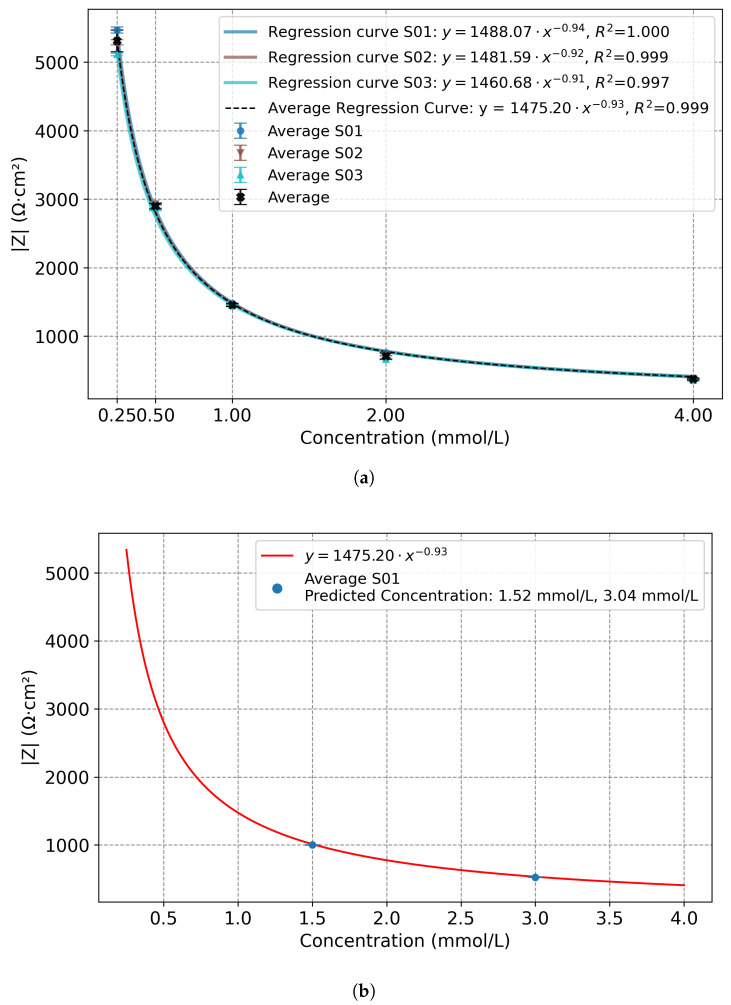
(**a**) Impedance modulus (|*Z*|) measured at 15.8 kHz as a function of concentration. Mean values and standard deviations are computed from three repeated measurements per concentration and per sensor. The regression curves for each sensor (S) follow a hyperbolic trend. In black: overall mean values, standard deviations, and the average regression curve computed on the whole dataset. (**b**) Regression model and impedance modulus values measured at 15.8 kHz for the 1.50 mmol/L and 3.00 mmol/L nominal solutions and estimated concentrations from sensor (S) S01.

Figure 5a presents the mean values of the impedance modulus (|*Z*|) as a function of concentration, along with the standard deviations, obtained from three repeated measurements for each concentration and each sensor. The figure also reports the overall mean values and standard deviations (in black), which were computed from the entire dataset for each concentration across 3 nominally identical sensors. The data clearly show an inverse relationship between electrolyte concentration and impedance modulus. Since the decrease is not constant across the concentration range but follows a progressively attenuated trend, the relationship was modeled using a non-linear power regression (Equation (3)).

(3) $$\;y=1475.20\;\cdot \;x^{-0.93}$$

In Equation (3), *y* represents the normalized impedance modulus, expressed in $Ω\;\cdot \;{\mathrm{cm}}^{2}$, and *x* denotes the KCl concentration in mmol/L.

Figure 5a also reports the computed regression curve for each sensor, and the average regression curve computed on the mean values of each concentration across the whole dataset of 3 sensors (black curve in Figure 5a).

Quantitatively, the impedance modulus decreases as the KCl concentration in solution increases, ranging from $\left(5300\pm 160\right)\;Ω\;\cdot \;{\mathrm{cm}}^{2}$ at 0.25 mmol/L to $\left(376\pm 14\right)\;Ω\;\cdot \;{\mathrm{cm}}^{2}$ at 4.00 mmol/L, corresponding to a reduction greater than 90% (the value after the symbol ± represents the standard deviation calculated across the three sensors for each concentration). These results confirm that interdigitated electrodes combined with high-frequency EIS provide an effective tool to monitor the salinity, and hence the conductivity, of solutions within the investigated range (0.25mmol/L÷4.00mmol/L). Moreover, the standard deviations computed on the whole dataset (considering the 3 sensors tested, and thus the 9 different measurements for each frequency) for each concentration were below 5% for all measurements, confirming both the good repeatability of the method and the reliability of the measurement system. This approach also demonstrates the feasibility of using a single-frequency measurement for rapid and quantitative monitoring of solution conductivity. Thus, based on these results, and after identifying the resistive region of the impedance spectra, the same single-frequency approach can be feasibly applied to sensors fabricated under identical printing and design conditions, which are expected to exhibit comparable morphological and electrical characteristics. This allows using a simpler measurement circuit, working without sweeping the test frequency.

To further validate the regression model, additional measurements were performed using the sensor S01 in two solutions with nominal concentrations of 1.50 mmol/L and 3.00 mmol/L. This sensor was selected among the others as representative, since the responses of the three sensors are mostly overlapped, as reported in Figure 5a.

The impedance modulus measured at 15.8 kHz was |Z| = $\left(525.5\pm 1.1\right)\;Ω\;\cdot \;cm^{2}$ and |Z| = $\left(1000.4\pm 2.2\right)\;Ω\;\cdot \;cm^{2}$ for the nominal concentration of 1.50 mmol/L and 3.00 mmol/L, respectively (the value after the symbol ± represents the standard deviation of the mean values from three repeated measurements performed with the sensor S01 for each concentration). Then, the corresponding concentration was estimated from the regression curve. As shown in Figure 5b, the regression model provided accurate identification of the unknown concentration values. The regression curve allowed us to predict the concentration of the solutions, with values of 1.52 mmol/L and 3.04 mmol/L. The differences between the nominal and predicted concentrations were +0.02 mmol/L (1.3%) for the 1.50 mmol/L solution and +0.04 mmol/L (1.3%) for the 3.00 mmol/L solution, confirming the robustness of the regression model. This represents a step forward towards real-world applications, where the electrolyte composition is not known a priori and reliable, rapid, and non-invasive diagnostic tools are required.

### 4.3. Electrochemical Impedance Measurements on Agar Samples

**Figure 6:**
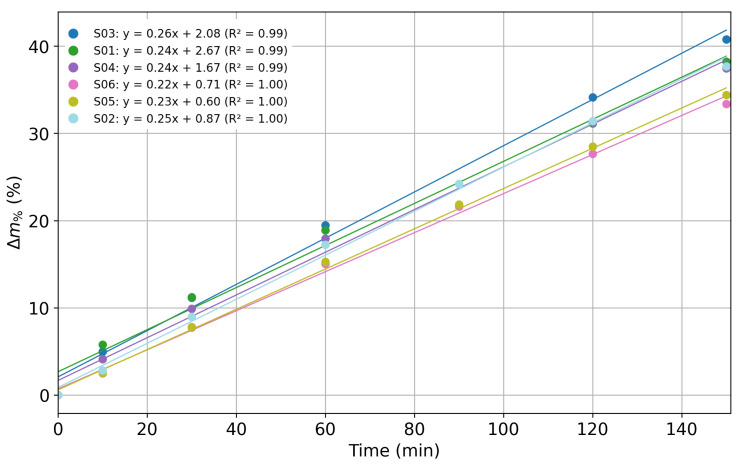
Percentage mass loss ($\Delta m_{\%}$) of agar samples as a function of exposure time in a climatic chamber with the corresponding linear regression curves (S: sensor).

The percentage mass losses of the agar samples as a function of time of exposure in the climatic chamber are reported in Figure 6.

It is worth noticing that in the experiments the ($\Delta m_{\%}$) exhibits a linear trend over time, with an average slope of (0.24±0.01) %/min (the value after the symbol ± represents the standard deviation of the slope values obtained from the individual regression analyses performed for each sensor), and an average regression coefficient $R^{2}\approx 0.99$. This confirms the suitability of the experimental setup for hydration sensor characterization, as the agar water content decreases linearly with time.

**Figure 7:**
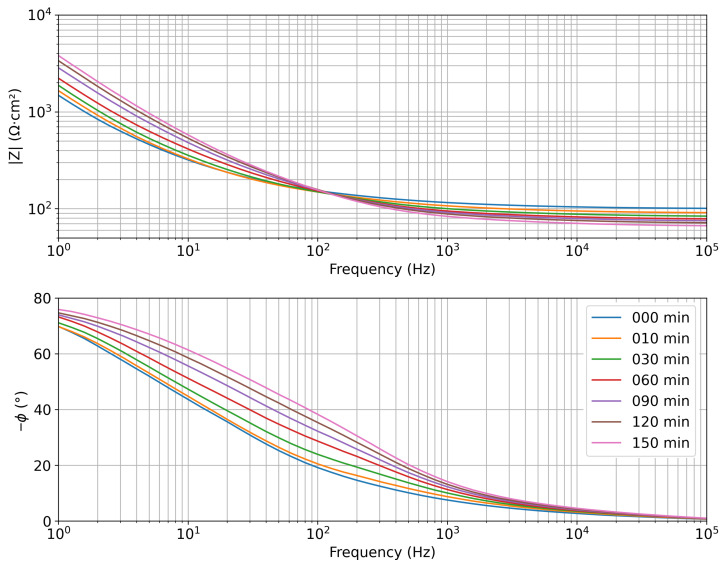
Representative impedance spectra (Bode plots) acquired on agar gel samples during dehydration, after 10, 30, 60, 90, 120, and 150 min of exposure in the climatic chamber.

Representative Bode plots (Figure 7) reveal a distinct dependency on time and hence mass loss, and thus hydration, of both impedance modulus and phase. At high frequencies (resistive region), the impedance modulus decreases consistently with the increase in ionic concentration inside the gel caused by water evaporation, so |*Z*| is inversely related to dehydration. As the water content decreases, the residual ionic density and conductivity increase, resulting in a reduction of resistivity. At low frequencies, the impedance modulus increases with evaporation, thus dehydration. However, as reported in [37,44], this part of the spectrum is significantly affected by electrode–sample interfacial effects, which are not directly related to hydration changes and are therefore less relevant for this study. The phase variation at mid-frequencies indicates a change in the capacitive–dielectric response of the agar gel during dehydration. As dehydration increases, the phase of the impedance increases, which can be interpreted as an enhancement of the capacitive effect due to reduced ionic mobility and changes in the dielectric properties of the medium [44].

It is worth noticing that, from the perspective of a future in vivo application, the use of phase values to predict the hydration levels of a patient is more convenient than the impedance modulus. Indeed, the latter is expected to have large inter-patient variability because, as shown in the previous measurements in saline solutions, it has a strong dependency on the conductivity of the investigated medium. So, finding a baseline value to compute hydration variation from the impedance modulus would be challenging. On the other hand, phase values are mainly affected by the shift from resistive to capacitive behavior in the different frequency ranges. For this reason, the Authors decided to perform the data analysis on impedance spectra acquired on agar samples, taking into consideration the phase values and not the impedance modulus.

**Figure 8:**
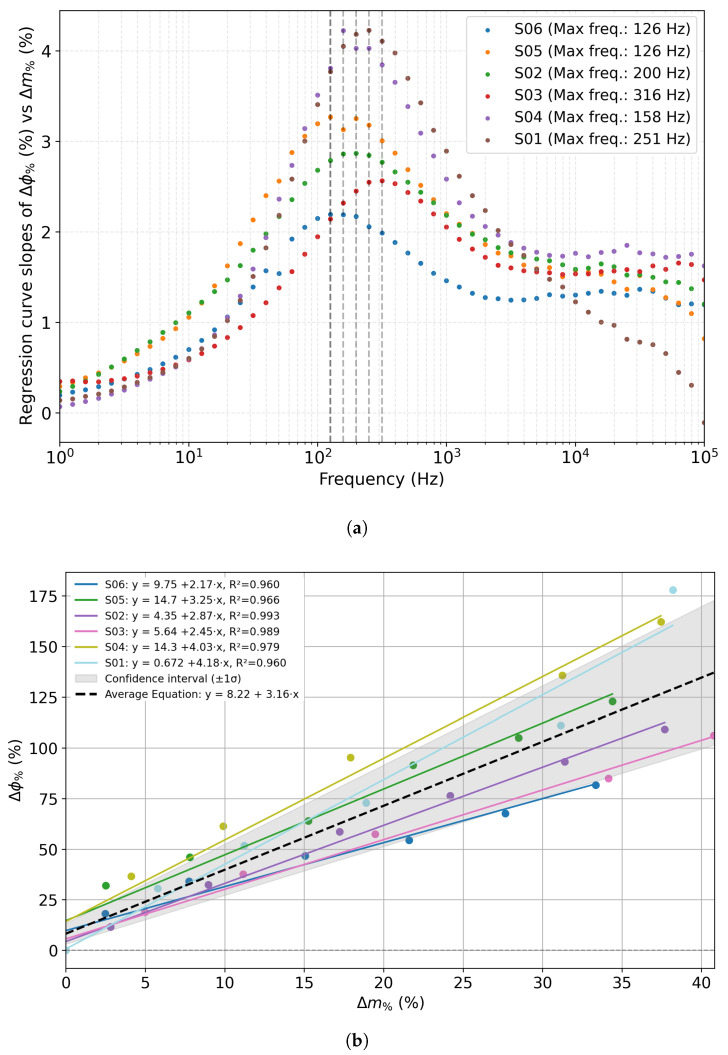
(**a**) Slopes of the regression curve between the phase shift $\Delta \phi_{\%}$ and the percentage mass loss $\Delta m_{\%}$ as a function of frequency for multiple sensors. (**b**) Phase shift $\Delta \phi_{\%}$ at 199.5 Hz as a function of percentage mass loss $\Delta m_{\%}$, with the corresponding regression curves for each sensor ( (solid lines; S: Sensor); the black dashed line represented the average regression curve; the shaded area is the ±σ confidence interval representing the standard deviation of the slopes across sensors.

Figure 8a shows, for each acquired frequency, the variable slope of the regression curve computed from the graph reporting the normalized phase shift ($\Delta \phi_{\%}$, as defined in Equation (2)) as a function of the percentage mass loss ($\Delta m_{\%}$, as defined in Equation (1)), for all investigated sensors.

A consistent trend is observed across the datasets: the slope increases with frequency, reaching a pronounced maximum in the range of approximately 125 Hz–316 Hz, before decreasing again at higher frequencies. This maximum slope indicates the frequency at which the phase response is most sensitive to mass loss, with individual sensors showing peak sensitivity at slightly different values (Figure 8a, Table 2). Overall, the results highlight a common frequency range where the correlation between phase change and water loss is strongest, suggesting that this range is optimal for dehydration monitoring applications. The average value of the frequency of maximum slope among sensors, i.e., 196.2 Hz, was selected as a diagnostic marker for comparison among sensors. Then, the closest available value in the frequency scan, i.e., 199.5 Hz, was selected to extract the $\Delta \phi_{\%}$ (as defined in Equation (2)) values for each sensor at each time point.

Figure 8b reports the $\Delta \phi_{\%}$ values at 199.5 Hz plotted as a function of the percentage mass loss for all investigated sensors. Each solid line corresponds to the linear regression obtained for a single sensor, while the black dashed line represents the average regression, computed as the mean of the individual slopes and intercepts across all sensors. The shaded area indicates the ±1σ confidence interval, where σ represents the standard deviation of the slope values across the six sensors. The results show a clear linear relationship between the relative phase variation and the mass loss, confirming that the percentage phase shift increases with dehydration. The average slope is (3.16±0.82)%/% (the value after the symbol ± represents the standard deviation of the slopes of the 6 sensors) per percent mass loss, with an average regression coefficient $R^{2}\approx 0.98$, and an average deviation% (deviation of each slope from the average slope value, expressed as a percentage) of 20.9%, as summarized in Table 3. The relative deviation of 20.9% in slope mainly reflects intrinsic morphological differences in the electrodes related to the inkjet printing process, together with possible local variations in the composition and ions distribution of the agar samples. Continued refinement of the sensors’ printing and agar gel preparation procedures in future work will enhance uniformity across devices.

Overall, the results demonstrate that printed interdigitated electrodes can detect changes in the water content of agar samples with both sensitivity and consistency, through variations in impedance modulus and phase. Specifically, the mid-frequency range proved to be the most informative for dehydration monitoring, with the phase variation at 199.5 Hz showing a clear correlation with water loss. This finding confirms that the sensing mechanism is consistent across the sensors produced with identical geometry and printing parameters. This reproducibility supports the feasibility of employing a representative single-frequency measurement for dehydration monitoring within this class of devices. Moreover, the same methodological approach can be extended to other sensor geometries or materials by first identifying the frequency region most sensitive to hydration-induced phase variations and then using it to extract quantitative information on hydration changes. The results suggest that, for future developments and applications, EIS measurements should focus on this frequency range to optimize sensitivity while reducing measurement complexity.

It is worth underlining that the tests evaluated the sensor response across a broad range of local water-content variations (0–40%) in agar gel samples, chosen to define the sensor operational boundaries beyond strictly clinical conditions. In this context, the mass-loss values $\Delta m_{\%}$ refer to relative water loss from the gel sample, and are therefore not directly comparable to whole-body mass loss in humans. Systemic dehydration in adults typically corresponds to a total body mass loss of about 2–3%, but this relationship is physiologically complex and non-linear due to water redistribution and electrolyte balance effects [47,48,49]. Furthermore, skin hydration is highly variable across individuals, anatomical sites, and measurement techniques, and depends on whether only the stratum corneum or deeper layers are considered. Literature reports approximate water-content ranges for healthy skin of 50–70%, with values below 40–50% associated with mild to severe dehydration and those above 80% with hyperhydration or edema [31,50,51,52]. Thus, the selected 0–40% relative local mass-loss interval effectively spans the transition from normal to dehydrated skin, providing a solid basis for assessing the sensor’s response across relevant hydration levels. Within the range tested in this work, the IDEs proved sufficient sensitivity, enabling the detection of subtle variations compatible with early dehydration levels. Future work will focus on in vivo validation to correlate impedance-derived metrics with physiological hydration markers, ultimately defining models and quantitative thresholds for clinical interpretation.

## 5. Conclusions

This work demonstrates the potential of inkjet-printed interdigitated electrodes as sensitive and reliable components for wearable hydration monitoring. The morphological analyses confirmed the sensor’s structural robustness and stability. EIS measurements in saline solutions established a predictive correlation between measured conductivity and salt concentration, leading to a regression model able to predict the concentrations of two solutions with an accuracy of about 1.3%. EIS experiments on agar gel phantoms validated the sensors’ ability to detect dehydration, establishing the phase shift at mid-frequencies as a key diagnostic parameter and identifying a robust linear relationship between impedance phase relative variation at 199.5 Hz and water loss, with consistent sensitivity across sensors. By assessing sensitivity, repeatability, and working frequency optimization, this study advances IDE-based sensing toward real-world applications in elderly care. These findings represent a fundamental step forward for the use of IDE sensors under more complex and realistic conditions, such as in vivo skin hydration monitoring. Future efforts will focus on in vivo validation and integration with wireless wearable systems to ensure comfort, robustness, and clinical applicability. In particular, since the polyimide-based IDE sensors are skin-conformable, they will be integrated into wearable patches with biocompatible adhesives and flexible interconnects, allowing direct electrical contact with the skin. Such a configuration would enable in vivo impedance-based hydration monitoring when placed on regions like the forearm or torso. Thus, integration into a multisensor device will enable continuous, non-invasive, and decentralized health monitoring, thereby contributing to more effective prevention of dehydration and improving healthcare practices.

## Figures and Tables

**Table 1:** Mean values and standard deviations of impedance modulus |Z| extracted at 15.8 kHz for KCl solution with different concentrations across all the datasets of 3 nominally identical sensors.

Concentration (mmol/L)	|Z| Mean (Ω·cm^2)^	Std. Dev. (Ω·cm^2^)
0.25	5300	160
0.50	2900	42
1.00	1456	20
2.00	711	43
4.00	376	14

**Table 2:** Maximum slopes of the regression curve between the normalized variation of phase angle $\Delta \phi_{\%}$ and the percentage mass loss as a function of frequency for all investigated sensors, together with the relative frequency, and the $R^{2}$ of the linear regression.

Sensor	Max. Slope (%/%)	Frequency (Hz)	*R* ^2^
S01	4.23	252	0.960
S02	2.88	200	0.993
S03	2.60	316	0.992
S04	4.22	158	0.974
S05	3.27	126	0.969
S06	2.20	126	0.963
Average	3.23	196.2	0.975

**Table 3:** Results of the linear regression between phase shift and percentage mass loss at 199.5 Hz for each sensor: slope, $R^{2}$ of the linear regression, and Deviation% (deviation of each slope from the average slope value), and average values.

Sensor	Slope (%/%)	*R* ^2^	Deviation% from Slope Average Value
S01	4.18	0.960	32.4
S02	2.87	0.993	9.2
S03	2.45	0.989	22.4
S04	4.03	0.979	27.5
S05	3.25	0.966	2.9
S06	2.17	0.960	31.2
Average	3.16	0.975	20.9

## Data Availability

Data available upon request to the authors.

## Abbreviations

The following abbreviations are used in this manuscript:

BIA	Bioelectrical Impedance Analysis
CE	Counter Electrode
EIS	Electrochemical Impedance Spectroscopy
IDE	Interdigitated sensor
MuSe	Multi-Sensor Wearable Device for Telemedicine
SEM	Scanning Electron Microscopy
WE	Working Electrode
